# Neuroinflammation in mild respiratory COVID-19: insights into cognitive impairment in milder cases

**DOI:** 10.1186/s40779-022-00431-x

**Published:** 2022-12-20

**Authors:** Qian Li, Chun Dang, Li-Hua Wang

**Affiliations:** 1 Department of Neurology, the Second Affiliated Hospital of Harbin Medical University, Harbin, 150081 China; 2 Department of Periodical Press, National Clinical Research Center for Geriatrics, West China Hospital, Sichuan University, Chengdu, 610000 China

**Keywords:** Coronavirus disease 2019 (COVID-19), Cognitive impairment, Neuroinflammation, Microglia, C-C motif chemokine ligand 11 (CCL11)

Severe acute respiratory syndrome coronavirus 2 (SARS-CoV-2) infection has been extensively shown to cause many neurological sequelae, and cognitive deficits (known as “brain fog”) may particularly and widely occur even in individuals with mild symptoms [1]. Peripheral hyperinflammation as well as central nervous system (CNS) local immune responses may synergistically contribute to brain autoimmune injury. In addition to the direct neuroinvasion of SARS-CoV-2 and nonimmune effects such as severe systemic hypoxemia and vascular thrombosis, the central mechanism by which SARS-CoV-2 accelerates cognitive-related symptoms may be related to immune effects [2]. However, the precise neuroinflammatory mechanisms of SARS-CoV-2 infection have not been fully established. Fernández-Castañeda et al. [3] provided direct evidence and unique insights into the potential mechanism of cognitive impairment in mild respiratory coronavirus disease 2019 (COVID-19) cases.

“Long COVID” has become another major public health crisis. The “lung-brain axis” has recently received increasing attention, and research published in *Nature* by Hosang et al. [4] suggested a close link between the lung microbiota and brain autoimmune responses, providing novel evidence that peripheral organs influence immune responses in the CNS. Inflammation has been considered to play a crucial pathogenic role and has been implicated in the neuropathological cascade leading to the development of cognitive dysfunction symptoms [5]. The inflammatory changes underlying SARS-CoV-2 post-infection that are associated with cognitive impairment, however, remain to be clarified. Fernández-Castañeda et al. [3] revealed that peripheral respiratory SARS-CoV-2 infection causes significant and persistent CNS inflammation. Different immune challenges may induce diverse immunological responses, COVID-19 and pandemic influenza have both common and specific immune features.

The peripheral cytokine storm initiated by SARS-CoV-2 infection may subsequently impact and trigger an inflammatory response in the nervous system [6]. Understanding the pathological mechanism of SARS-CoV-2 affecting the nervous system is critical for selecting potential therapeutic targets for patients. Microglia are sensitive to even minor changes in the microenvironment within the CNS and may trigger subsequent inflammatory cascades, leading to secondary immune injury. Fernández-Castañeda et al. [3] demonstrated that the neuroinflammation caused by the mild respiratory SARS-CoV-2 infection may be a link between mild respiratory COVID-19 and cognitive impairment. The CNS pathological mechanisms may be caused by the elevation of C-C motif chemokine ligand 11 (CCL11) associated with COVID-19, which exhibited similar neuropathology caused by cancer therapy. Neurotoxic CCL11 causes white matter selective microglial reactivity, then elevated CCL11 and active microglia lead to deleterious cascade effects on CNS multi-lineage neuronal cellular function and structure dysregulation, particularly on oligodendrocytes, myelinated axons, and hippocampal neurogenesis, which in turn causes the cognitive dysfunction symptoms in COVID-19 (Fig. 1).

**Fig. 1:**
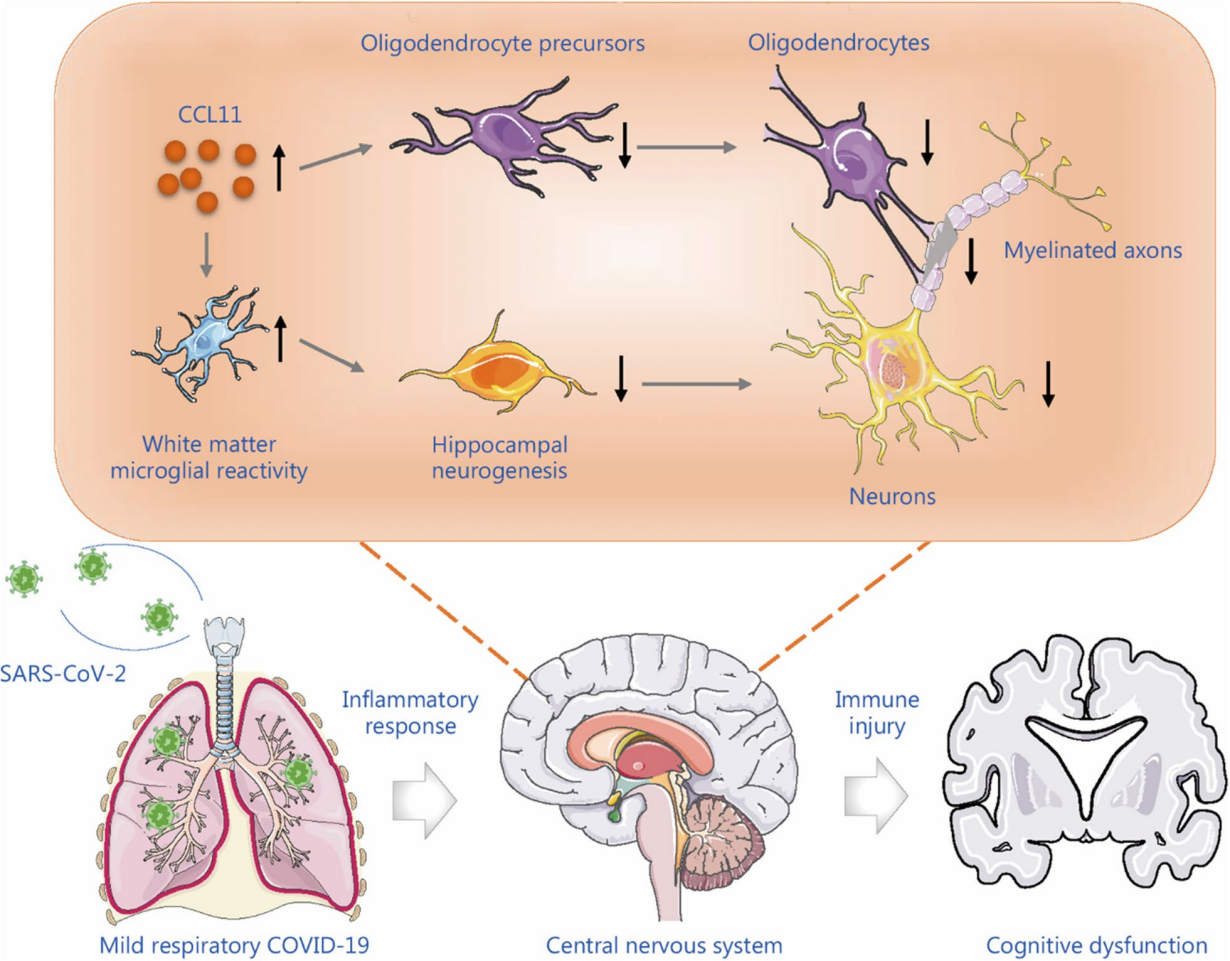
Neuroinflammation is the central pathophysiology that links mild respiratory COVID-19 to cognitive impairment. Respiratory SARS-CoV-2 infection can cause an excessive peripheral inflammatory response to result in the consequent immune injury in CNS. The elevated CSF CCL11 and white matter microglial reactivity are associated with multi-lineage cellular dysregulation in the CNS, including impaired hippocampal neurogenesis, persistent loss of oligodendrocytes, and myelinated axons. SARS-CoV-2 severe acute respiratory syndrome coronavirus 2, COVID-19 coronavirus disease 2019, CCL11 C-C motif chemokine ligand 11, CNS central nervous system, CSF cerebrospinal fluid

Taken as a whole, Fernández-Castañeda et al. [3] demonstrated that cognitive impairment following mild respiratory COVID-19 is associated with persistent neuroinflammation, providing robust animal and human data to illustrate the pathophysiological similarities between “COVID fog” and “chemo fog” syndromes. As such, anti-inflammatory strategies targeting microglia or CCL11 signaling may hopefully prevent and correct this multicellular dysregulation. However, the underlying precise regulatory mechanism and signal transduction mediating respiratory infection-induced microglial reactivity in subcortical white matter remain to be fully elucidated. The mechanisms of cognitive impairment in the context of other causes, such as ageing and stroke, might provide further insight into the mechanisms of COVID-19-related cognitive deficits. Moreover, given that the role of microglia is very complicated and can be both neuroprotective and neurotoxic, the determination of the key regulators that can convert microglia into an anti-inflammatory phenotype or regulate the initiation of microglial activity is urgently needed, as the manipulation of these regulators may have the ability to prevent or alleviate neuropathology and improve impaired cognitive performance. Nevertheless, additional studies should be performed to investigate the immunomodulatory effects of peripheral infiltrated immune cells, other subtypes of brain immune cells such as astrocytes, and cellular neuroimmune interactions between the CNS and peripheral systems [7].

An analysis of 2-years retrospective cohort study indicated that the increased cognitive deficit after COVID-19 persisted throughout the follow-up [8]. Important questions remain that whether this pathology is reversible or permanent. Furthermore, it requires to be fully determined whether neuroimmune injury follows COVID-19 caused by novel SARS-CoV-2 variants, such as Omicron variants, or breakthrough infections in vaccinated individuals, as well as whether infection with the SARS-CoV-2 Omicron variant in children could cause unique neuropathology. Therefore, studies and comparisons of patients in various mentioned situations with long-term clinical outcomes will be highly valuable.

## Data Availability

Not applicable.

## Abbreviations

SARS-CoV-2: Severe acute respiratory syndrome coronavirus 2
COVID-19: Coronavirus disease 2019
CNS: Central nervous system
CCL11: C-C motif chemokine ligand 11
